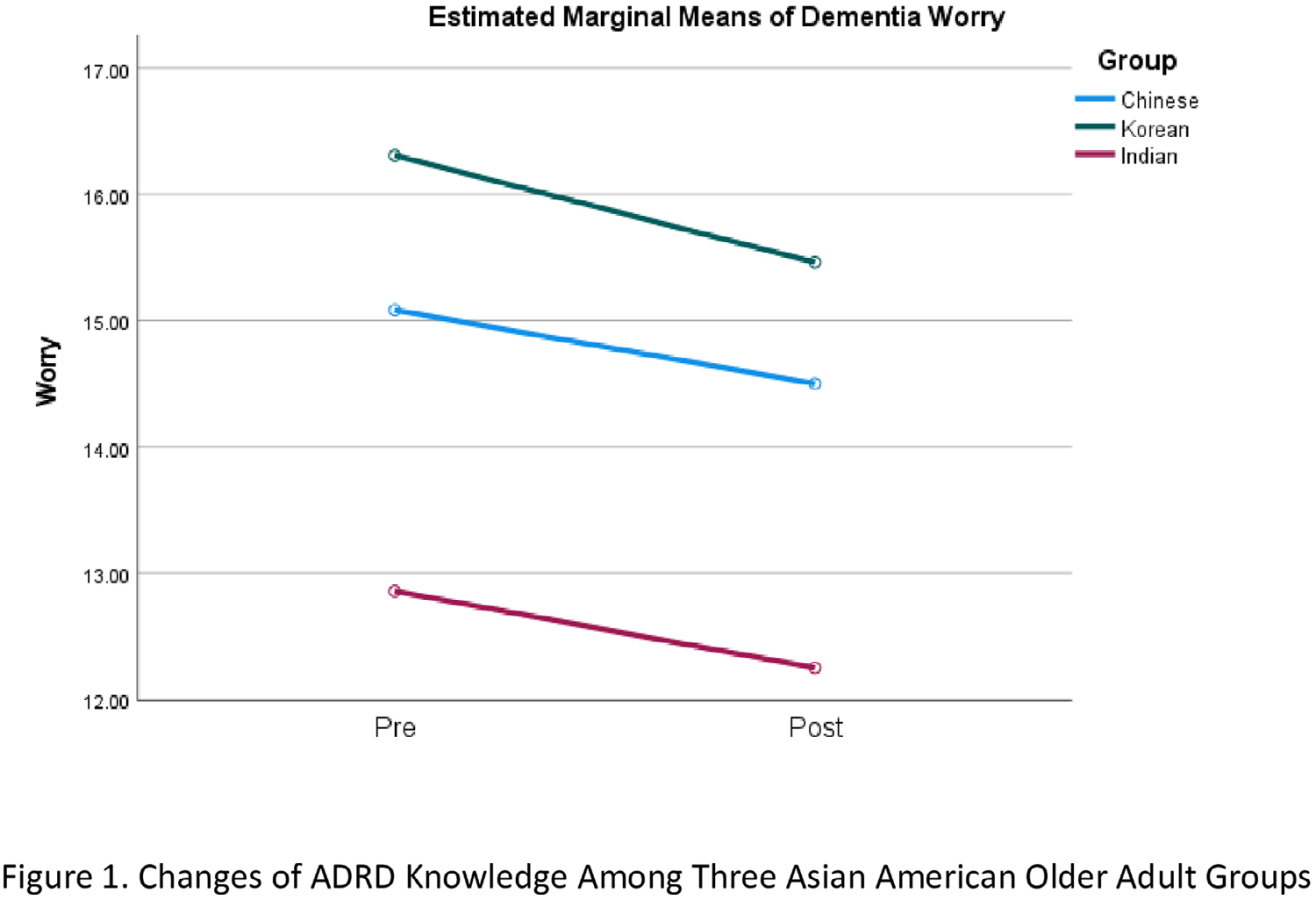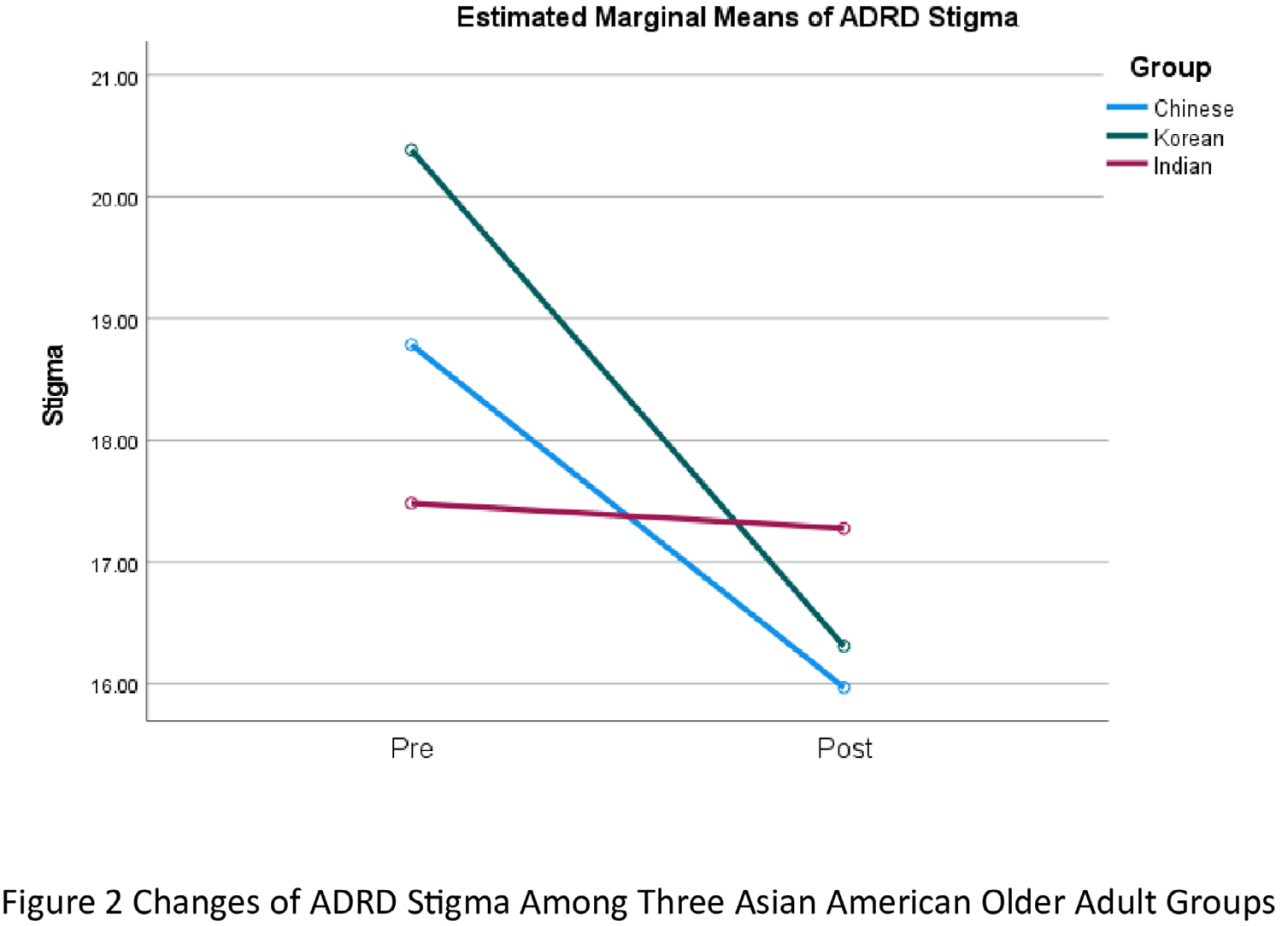# Effects of A Culturally Tailored ADRD Education Program for Asian American Older Adults in Michigan

**DOI:** 10.1002/alz70858_107770

**Published:** 2025-12-26

**Authors:** Fei Sun, Daniel Velez Ortiz

**Affiliations:** ^1^ Michigan State University, East Lansing, MI, USA

## Abstract

**Background:**

Alzheimer's disease and related dementias (ADRD) affect approximately 202,800 residents in Michigan. Older Asian Americans often face limited knowledge, culturally biased beliefs, and elevated worries regarding ADRD, yet few culturally appropriate programs exist to address these needs in Michigan.

**Methods:**

This study evaluated a newly developed intervention aimed to enhance ADRD knowledge and reduce stigma and worry among three older Asian Americans (i.e., Chinese, Indian, Korean). The intervention consisted of an educational session and one chosen culturally appropriate activity, with input from ethnic community stakeholders. Culturally tailored activities included ethnic meals for the Chinese group, mindfulness yoga for the Indian group, and Tai Chi for the Korean group. Participants were recruited through senior centers, senior housing facilities, religious congregations, ethnic organizations, and self‐referrals. Each ethnic group received pre and posttest evaluations.

**Results:**

Outcome measures consisted of ADRD knowledge, stigma, and dementia worry. The intervention was provided to 15 Korean participants (Mage = 76.4/ SD = 10.0, 80% female), 82 Chinese participants (Mage = 82.2/SD = 6.6, 60% female), and 32 Indian participants (Mage = 74.4/SD = 7.3, 56.3% female). Korean participants showed significant increases in ADRD knowledge (Hedges’ g = 0.60, *p* < .05) and reductions in stigma (Hedges’ g = ‐1.00, *p* < .01), though changes in worry were not significant. Chinese participants experienced similar improvements in knowledge (Hedges’ g = 0.32, *p* < .01) and stigma (Hedges’ g = ‐0.45, *p* < .01). Indian participants had significant gains in knowledge (Hedges’ g = 0.50, *p* < .01), but changes in stigma and worry were not statistically significant. Figures 1 and 2 illustrate changes in ADRD knowledge and stigma across the groups.

**Conclusions:**

This study underscores the value of culturally tailored, community‐based interventions for improving ADRD literacy among older Asian Americans. Knowledge improved across all groups, while stigma reduction was observed in Korean and Chinese participants. Addressing dementia‐related worry remains challenging, calling for innovative strategies to manage emotional responses, such as anxiety and fear, related to ADRD awareness. Further research is needed to understand subgroup differences in dementia stigma reduction.